# Habitat and Scale Shape the Demographic Fate of the Keystone Sea Urchin *Paracentrotus lividus* in Mediterranean Macrophyte Communities

**DOI:** 10.1371/journal.pone.0035170

**Published:** 2012-04-20

**Authors:** Patricia Prado, Fiona Tomas, Stefania Pinna, Simone Farina, Guillem Roca, Giulia Ceccherelli, Javier Romero, Teresa Alcoverro

**Affiliations:** 1 Institut de Recerca i Tecnología Agroalimentàries (IRTA), Aquatic Ecosystems, Tarragona, Spain; 2 Dept. of Biology East Carolina University, Greenville, North Carolina, United States of America; 3 Instituto Mediterráneo de Estudios Avanzados (IMEDEA), Islas Baleares, Spain; 4 Dept. of Science for Nature and Environmental Resources University of Sassari, Sassari, Italy; 5 Centre d'Estudis Avançats de Blanes (CEAB)-CSIC, Girona, Spain; 6 Departamento de Ecología, Facultad de Biología, Universidad de Barcelona, Barcelona, Spain; University of Canterbury, New Zealand

## Abstract

Demographic processes exert different degrees of control as individuals grow, and in species that span several habitats and spatial scales, this can influence our ability to predict their population at a particular life-history stage given the previous life stage. In particular, when keystone species are involved, this relative coupling between demographic stages can have significant implications for the functioning of ecosystems. We examined benthic and pelagic abundances of the sea urchin *Paracentrotus lividus* in order to: 1) understand the main life-history bottlenecks by observing the degree of coupling between demographic stages; and 2) explore the processes driving these linkages. *P. lividus* is the dominant invertebrate herbivore in the Mediterranean Sea, and has been repeatedly observed to overgraze shallow beds of the seagrass *Posidonia oceanica* and rocky macroalgal communities. We used a hierarchical sampling design at different spatial scales (100 s, 10 s and <1 km) and habitats (seagrass and rocky macroalgae) to describe the spatial patterns in the abundance of different demographic stages (larvae, settlers, recruits and adults). Our results indicate that large-scale factors (potentially currents, nutrients, temperature, etc.) determine larval availability and settlement in the pelagic stages of urchin life history. In rocky macroalgal habitats, benthic processes (like predation) acting at large or medium scales drive adult abundances. In contrast, adult numbers in seagrass meadows are most likely influenced by factors like local migration (from adjoining rocky habitats) functioning at much smaller scales. The complexity of spatial and habitat-dependent processes shaping urchin populations demands a multiplicity of approaches when addressing habitat conservation actions, yet such actions are currently mostly aimed at managing predation processes and fish numbers. We argue that a more holistic ecosystem management also needs to incorporate the landscape and habitat-quality level processes (eutrophication, fragmentation, etc.) that together regulate the populations of this keystone herbivore.

## Introduction

The population dynamics of keystone species can have far-reaching consequences. Population outbreaks, particularly of herbivores, have been observed to cause important ecosystem shifts in terrestrial, freshwater, and marine environments [1]–[3]. While top-down factors like predation are often strong enough to explain population dynamics in a multiplicity of ecosystems [4], when a species has a life history that spans multiple spatial scales and habitats, it is often difficult to explain such dynamics with a single factor. This is particularly true in the case of marine benthic organisms with planktonic larval stages, which depend both upon factors controlling the arrival of new individuals and on the structural and functional properties of the habitats in which they recruit [5]–[6]. For these types of organisms, the identification of the population bottlenecks provides crucial information, since it gives an indirect clue of where the potential limits and controls are acting in the life history of a species.

In understanding the demography of a marine keystone species, it is important to determine bottlenecks not merely in benthic life stages but also to recognize the “lost period” in the pelagic stage as well, since variation at this stage could be very important in determining settlement and recruitment [7]–[8]. The majority of studies focus on what processes influence a specific stage of the species (e.g., predation pressure, migration, and competition) but few have simultaneously considered the entire life cycle including planktonic and benthic stages [9]. The absence of studies that include all life-stage processes is in part a result of the absence of studies that include different scales [10]. In fact, most processes affecting different life-stages are scale-dependent, and the identification of certain, prevailing mechanisms for population control will depend on the study's spatio-temporal scale.

Since benthic stages are commonly sessile or territorial they are typically influenced by processes occurring at smaller spatial scales [11]–[12], including habitat-related processes, whereas dispersal of planktonic larvae is influenced by processes occurring at larger spatial scales [13]. Planktonic stages can be passively transported over thousands of kilometers [14]–[15] and colonize remote locations subjected to large-scale oceanographic phenomena [16]–[17]. Offshore processes, together with regional patterns of temperature and salinity, play an important role in determining the abundance and bio-geographical patterns of populations [18]. However, more specific factors, either abiotic (e.g., topography, prevailing winds) or biotic (e.g., adult abundance and fertility, planktonic predation pressure) may also control larval abundance at local scales [5], [10], [19]–[21]. Once the organism has transitioned into a benthic stage, variability in the abundance of adult populations might be again explained by large-scale differences in recruitment success [22] or by the absence of local predation control [23], but also by other factors such as habitat features and availability, landscape connectivity or resource distribution. All these factors, including their interplay, can be critical bottlenecks reducing the abundance of settlers and early post-settlement stages from hundreds or thousands to a few individuals per square meter [7], [24]–[26].

In the Mediterranean Sea, the sea urchin *Paracentrotus lividus* has been clearly recognized as a keystone herbivore due to its ability to transform macroalgal-dominated communities into barren areas characterized by increased cover of bare substrates and encrusting coralline algae, reduced biodiversity and altered ecosystem functions [27]. This sea urchin displays considerable variation in the abundance and size distribution of individuals among regions, sites and habitats (see review by [28]), which in turn leads to differential impacts by location. Sea urchin abundance and the expansion of those barrens have been linked to the overfishing of predatory fish species. Therefore, one of the keys to understanding the transition from erect algal communities to barrens is the regulation of sea urchin population dynamics [29]–[30]. In neighboring ecosystems dominated by the seagrass *Posidonia oceanica*, *P. lividus* also plays a central role by directly removing plant biomass, inducing nutrient export, and modifying plant production and reproduction [31]–[35].

Here, we attempt for the first time a study encompassing the whole life cycle of a sea urchin using an approach at different spatial scales. We examine the degree of coupling/uncoupling between the abundances of benthic and pelagic life-stages of the sea urchin *P. lividus* in order to identify the main life-history bottlenecks and further understand the role of habitat type in that coupling. We used a hierarchical sampling design at different spatial scales (100 s, 10 s and 1 km) and habitats (seagrass and rocky macroalgal beds) to describe the spatial patterns in the abundance of different demographic stages (i.e. larvae, settlers, recruits and adults) and elucidate the processes driving those patterns.

## Materials and Methods

### Study sites

**Figure 1:**
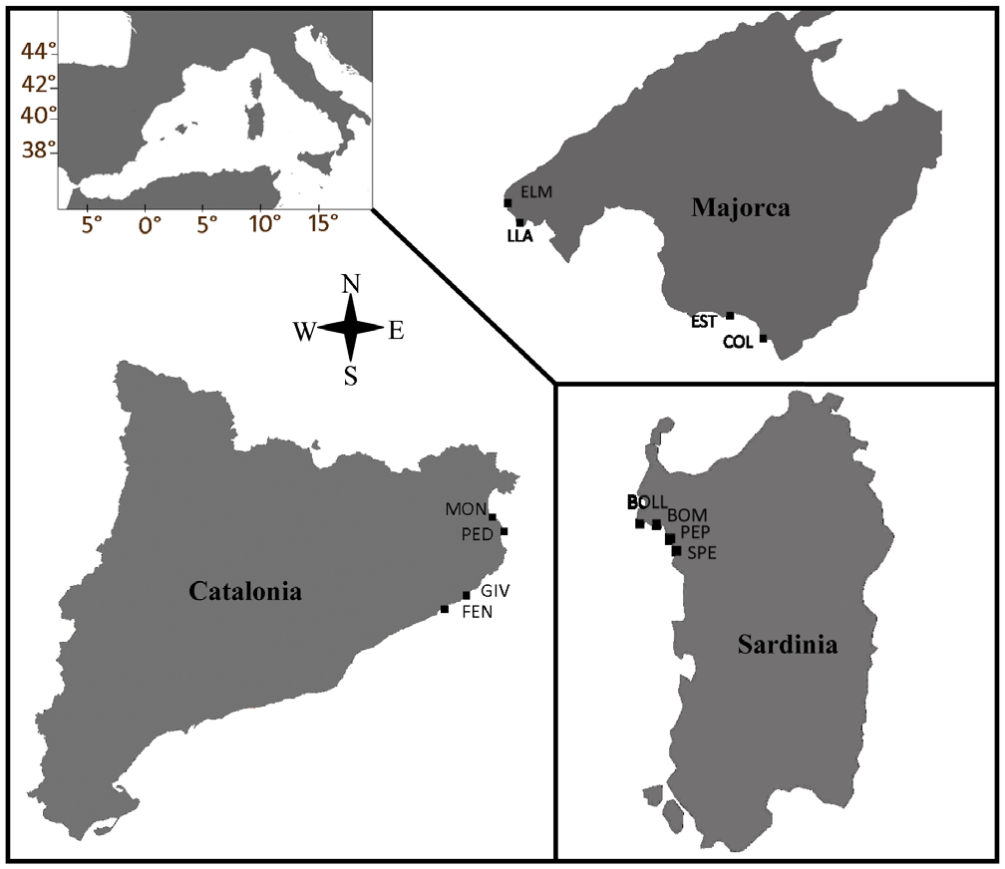
Map of the Western Mediterranean showing the three study regions and corresponding sites. In Catalonia: Montgó (Mon: 42°6.2′N; 3°10.1′E), Isla Pedrosa (Ped: 42°4.2′N; 3°12.1′E), Giverola (Giv: 41°44.1′N; 2°57.1′E), and Fenals (Fen: 41°41.3′N; 2°49.6′E); in Sardinia: Torre Bollo (Boll: 40°34.1′N; 8°9.5′E), Bombarde (Bom: 40°34.5′N; 8°15.4′E), Pepino (Pep: 40°32.2′N; 8°19.3′E), and Sperança (Spe: 40°29.4′N; 8°21.6′E); and in Majorca: Còlonia (Col: 39°18.5′N; 2°59.2′E), Estanyol (Est: 39°21.2′N; 2°54.6′E), St. Elm (Tel: 39°34.5′N; 2°20.5′E), and Cala Llamp (Lla: 39°31.5′N; 2°23.2′E).

The study was conducted within three distinctive regions of the NW Mediterranean Sea – NE of Iberian Peninsula (Catalonia), Majorca Island and Sardinia Island. These represent a regional scale, with regions separated by hundreds of kilometers (Fig. 1). Within each region, we selected 4 sites separated by tens of kilometers, which represent a medium scale (see site coordinates in Fig. 1). Sites were sampled in two zones that were 50 to 300 m apart and represent the local scale. Each zone included areas of seagrass habitat dominated by *Posidonia oceanica* and immediately adjacent rocky macroalgal habitat. The deployment of collectors for settlers, and abundance quadrates for recruits and adults (see later), were conducted at 5 m depth, according to documented maximum sea urchin abundance and herbivory pressure in shallow seagrass and macroalgal habitats [33], [36]–[37].

### Larvae

Planktonic larvae were sampled at each study zone on four consecutive occasions (spaced by 15 days) from late April to late June in Majorca and from mid May to early July 2008 in Catalonia and Sardinia (i.e., ca. 1.5 months). The rationale for this approach was to have the certainty that the larval peak would be captured and that the number of larvae present in each zone could be compared across spatial scales. The slight temporal lag in sampling across regions was considered to accommodate reported temporal differences in water temperature of ca. 2°C among regions [38]–[39] that appear to trigger the release of larvae by *Paracentrotus lividus* around late spring, when sea surface temperature is ≤18°C [24], [40]. Sampling was conducted by towing a plankton net at the sea surface, just above our study zones (see Fig. 1 for coordinates) and at a distance from shore that varied between 20 and 50 m. Each sampling event consisted of three replicate tows of 5 minutes (at a constant speed of 1.5 knots) conducted horizontally on the water surface using a 0.3 m diameter, 100 µm mesh net with a 10 cm diameter cod end jar. The total volume of water filtered at each tow (i.e., the product of tow distance (i.e., boat speed x time) by the mesh mouth area) was 56.83 m^3^. The entire samples were preserved in 250 ml containers and stored with 4% formalin in seawater buffered with excess sodium borate. In the laboratory, formalin was rinsed off and samples were sorted for total number of larvae (i.e. all larval stages) under a dissecting microscope. Samples from the Catalan coast, which contained very high densities of zooplankton or diatoms, were sub-sampled with a plankton splitter, while those from the other sites were examined as a whole. The number of larvae found at each time (n = 4) and tow (n = 3) resulted in n = 12 samples per zone and a total of 288 observations (12×2 zones ×4 sites ×3 regions) for the 3-way ANOVA analysis (see later). Data were then expressed as number of larvae per m^3^.

### Settlers

At each study zone, three replicate collectors consisting of scrub brushes with vegetal bristles were haphazardly deployed within the seagrass canopy and over the rocky bottom (see details in [36]). Collectors were deployed in the field for two 2-week periods between May and July in an attempt to capture the majority of the settling peak [24], [36], [40]. Each collector was removed after an initial 2-week period (i.e., settlers were less than 15 d old), replaced by a new collector, and transported to the laboratory within an icebox. Once in the laboratory, collectors were rinsed with high-pressure water through a 250 µm mesh and filtered material was preserved with alcohol 70% within glass containers for further sorting and counting under a dissecting microscope [40]. Replicate samples of settlers were calculated by adding the number of individuals obtained per collector at each of the two sampling occasions (i.e., resulting in three replicates per zone and habitat). Since sea urchin settlement is strongly linked to temperature [40], [41], we also calculated weekly sea surface temperature (SST) for each region using data for May–June from 1993 to 2007 available from the WDC-RSAT web site [42].

### Recruits and adults

At each zone (ca. 250 m^2^) and both habitats, SCUBA divers counted individual *P. lividus* within 15 haphazardly placed quadrats (50×50 cm), distant by ∼2 m and measured test diameters using calipers (precision of 0.5 cm). Given the low seasonal variability reported for seasonal densities of *Paracentrotus lividus* [34], [38], sampling of recruits and adults was conducted only once at each locality during the summer period. Sea urchins were grouped into size classes, which ranged from 0.5 to 8 cm. Individuals <3 cm in size were classified as recruits and those >3 cm were adults [43].

### Data analyses

**Figure 2:**
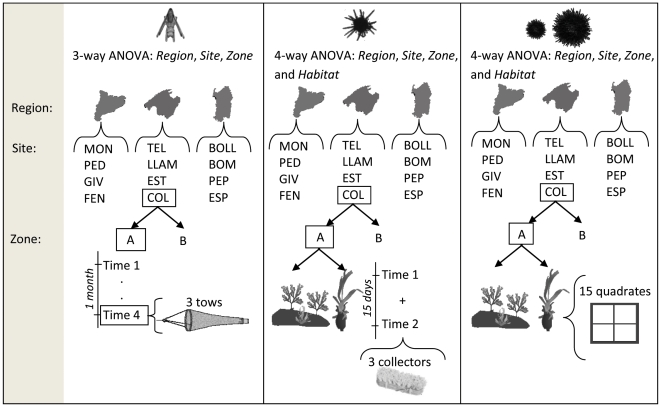
Hierarchical sampling design conducted to evaluate spatial variance of larval, settling, recruit and adult stages of *Paracentrotus lividus*. For each life-stage, the methodology used and the periodicity (larvae and settlers) is indicated. Note that for larvae and settlers, sampling times are added and not treated as a factor; for details see the Materials and Methods section. Site abbreviations as in Fig. 1.

The relationship between one life-stage and the following stage was analyzed using regressions, separately for seagrass and rocky macroalgal habitats. Differences in SST and in the number of weeks with SST ≤18°C among study regions was investigated with a one-way ANOVA with Region (three levels) treated as a random factor. Differences in the abundance of larvae were analyzed with a hierarchical three-way ANOVA design (Region, Site and Zone; all random factors) whereas the abundance data for settlers, recruits and adults were analyzed using four-way ANOVAs, with Habitat fixed and orthogonal, and Region, Site, and Zone random and hierarchical factors (see Fig. 2 for detailed differences in the sampling design among life-stages). For all ANOVAs, data were first tested for normality (Chi-square) and homogeneity of variances (Cochran's test). Data were transformed when necessary to satisfy ANOVA assumptions as indicated in the results section (Table 1). In some cases, however, assumptions could not be met even after transformation and the level of significance was fixed at α = 0.01 to minimize the probability of making a type II error [44]. We estimated variance components to further examine the contribution of each spatial-scale (region, site, zone, and replicate) to the total observed variation for each habitat. We thus first carried out new analyses of variance for each life-stage (except larvae) independently for seagrass and rocky macroalgal habitats. Variance calculations were then conducted by equating observed Mean Squares (MS) associated with replicate effects (i.e., the residual variation), Zone, Site, and Region to the expected MS [44] as indicated in Table 2.

**Table 1:** Hierarchical ANOVAs for spatial differences at each sea urchin life-stage.

a) Larvae	df	MS	F	p
Region = R	2	15261.19	9.61	**0.0058**
Site = S(R)	9	1589.58	2.53	0.0682
Zone = Z(R(S))	12	627.61	1.29	0.2253
Residual	264	487.25		
Transf: none; *C* = 0.37; p<0.01				

b) Settlers	df	MS	F	p
Habitat = H	1	1.58	1.32	0.3695
Region = R	2	170.81	141.50	**0.0000**
Site = S(R)	9	1.20	2.14	0.1091
Zone = Z(S(R))	12	0.56	2.63	**0.0044**
H×R	2	1.20	2.18	0.1685
H×S(R)))	9	0.55	3.94	**0.0151**
H×Z(S(R))	12	0.13	0.65	0.7927
Residual	96	0.21		
Transf: ln (x+1); *C* = 0.12 (ns)				

c) Recruits	df	MS	F	p
Habitat = H	1	546.01	1724.26	**0.0006**
Region = R	2	44.01	0.55	0.5940
Site = S(R)	9	79.70	4.67	**0.0078**
Zone = Z(S(R))	12	17.08	2.32	**0.0065**
H×R	2	0.31	0.01	0.9905
H×S(R)))	9	33.15	2.84	0.0477
H×Z(S(R))	12	11.68	1.58	0.0905
Residual	912	0.21		
Transf: none; *C* = 0.13; p<0.01				

d) Adults	df	MS	F	p
Habitat = H	1	12.91	0.85	0.4549
Region = R	2	161.86	17.95	**0.0007**
Site = S (R×H)	9	9.01	1.54	0.2372
Zone = Z (R×S×H)	12	5.83	8.76	**0.0000**
H×R	2	15.27	1.95	0.1974
H×S(R)))	9	7.81	3.93	**0.0152**
H×Z(S(R))	12	1.98	2.98	**0.0004**
Residual	912	0.66		
Transf: ln (x+1); *C* = 0.04 (ns)				

a) larvae; b) settlers; c) recruits (size <3 cm); and d) adults (size >3 cm). Statistically significant results (p<0.01 for non-transformable data) are indicated in **bold**. *C* = Cochran's C.

**Table 2:** Estimates of spatial variance at each sea urchin life stage.

		Variance							
		Larvae	Settlers		Recruits		Adults		
Source	MS estimates		P	R	P	R	P	R	
R	σ^2^_e_+nσ^2^_Z(S(R))_+znσ^2^_S(R)_+sznσ^2^_R_	142.4	487.1	13220.0	0.1	0.0	14.7	4.4	σ^2^_region_
S(R)	σ^2^_e_+nσ^2^_Z(S(R))_+znσ^2^_S(R)_	40.0	164.7	55.8	0.2	1.5	5.7	3.1	σ^2^_site_
Z(S(R))	σ^2^_e_+nσ^2^_Z(S(R))_	11.7	0	151.7	0.1	0.6	35.8	1.5	σ^2^_zone_
Residual	σ^2^_e_	142.4	69.3	207.3	1.9	12.8	35.1	16.9	σ^2^_replicate_

Components of variance for MS estimates and variances associated to each source of variation for larvae: s = 4, z = 2, n = 12; for settlers: s = 4, z = 2, n = 3; and for recruits and adults: s = 4, z = 2, n = 20. P = *Posidonia oceanica* seagrass meadows and R = rocky macroalgal habitats. Larvae data have not habitat associated to habitat because they were collected in the water column.

## Results

### Life stage abundances at the different spatial scales and habitats

**Figure 3:**
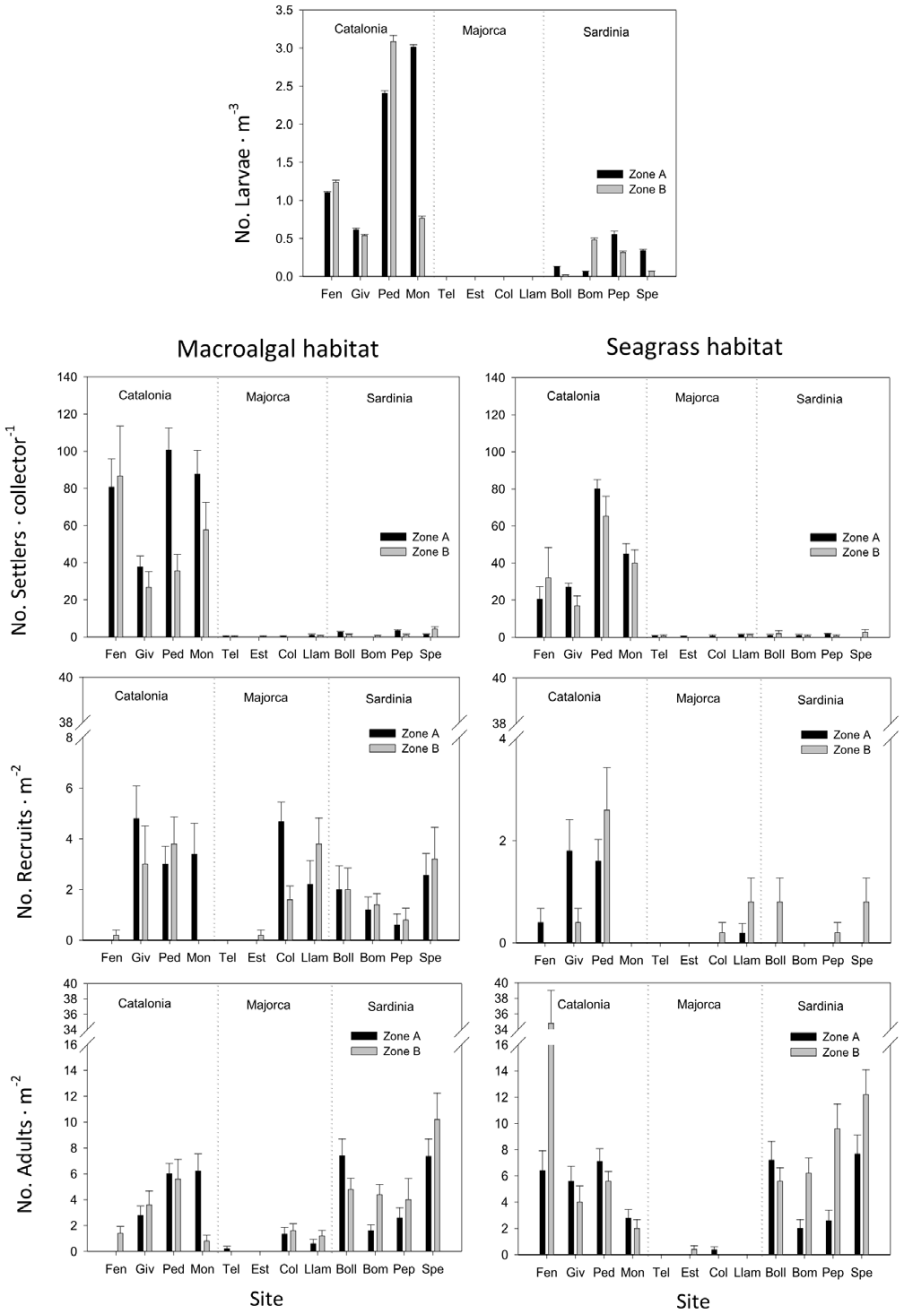
Abundance of sea urchin life-stages per habitat. Values show numbers of larvae (No. m^−3^), settlers (No. collector^−1^), recruits (sizes <3 cm; ind. m^−2^), and adult individuals (sizes >3 cm; ind. m^−2^) in rocky macroalgal and seagrass habitats at each spatial scale (region, site and zone). Site abbreviations as in Fig. 1.

Larval abundance was ∼6.6-fold higher in Catalonia with 1.59±0.044 larvae m^−3^ (mean ± SE) compared to Sardinia with 0.24±0.011 larvae m^−3^ and higher than in Majorca, where no larvae were found (Fig. 3, Table 1). In fact, most of the variance was observed at the regional level (100 km scale), and was much lower between sites (10 km scale) and zones (<100 m scale; see Tables 1–2, Fig. 3). Following the larval pattern, settler abundances were higher in Catalonia than in the other two regions, with no significant differences between habitats (Table 1). Mean values of settlers in Catalonia were 40.8±7.8 (ind • collector^−1^) in seagrass and 64.1±10 in rocky macroalgal habitats, in Sardinia they were 1.2±0.3 in seagrass and 1.8±0.5 in rocky macroalgal habitats and in Majorca they were 0.62±0.18 in seagrass and 0.37±0.11 in rocky macroalgal habitats. In both habitats, the effect of the regional scale (i.e. differences among regions) was again the major source of variance (see Table 2). On the other hand, the type of habitat appeared to play an important role in determining later demographic stages in both rocky macroalgal and in seagrass habitats. The abundance of recruits was ca. 4.2 times higher in rocky macroalgal habitats than in seagrass habitats (Fig. 3). However, despite low recruitment in seagrass habitats, adult abundances were typically higher than on nearby rocky macroalgal habitats (Fig. 3). Scale again interacted with these habitat processes to shape populations at each demographic stage (Table 1). Thus, while recruit abundance variability was highest at the medium scale (10 km) in both habitats (Tables 1–2), adult numbers varied the most at the regional-scale in rocky macroalgal habitats and at the local scale (less than 1 km) in seagrass habitats (Table 1 and 2). In both habitats, replicates represented an important source of variance in recruit and adult stages (Table 2).

### Coupling/uncoupling between life stages

**Figure 4:**
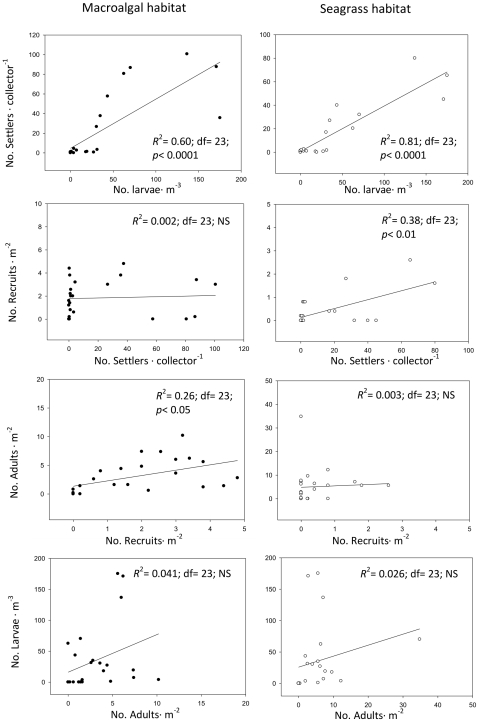
Regression analyses between successive life-stages at each habitat. Significant determination coefficients (*R* ^2^) are indicated (i.e., p<0.05).

No relationship was observed between larvae and adults when all regions where included, and the same lack of a relationship occurred for both habitats (Fig. 4). In contrast, there was a strong relationship between larval numbers and the abundance of the next life history stage (settlers) in both seagrass and rocky macroalgal habitats (Fig. 4).

Recruit numbers in rocky macroalgal habitats were unrelated to settler abundances, but adult abundance seemed weakly (although significantly) associated with recruit abundance (Fig. 4). In contrast, in seagrass beds the number of settlers was a reliable predictor of recruits' abundance, which was very low in all seagrass meadows surveyed (Fig. 3). Despite low recruitment, adult abundances in seagrass habitats were much higher than on nearby rocky macroalgal habitats (Fig. 3), resulting in a clear decoupling between recruit and adult populations in the seagrass habitat (Fig. 4).

### Between-region predictors

Sea Surface Temperature (SST) in June was significantly different among regions (one-way ANOVA: *F*_2,42_ = 80.54, *p*<0.0001). Higher values (in °C, mean ± SE) were registered in Majorca (23.40±0.19) than in Sardinia (22.35±0.25) and the Catalan coast (19.65±0.20). In addition, the number of weeks with SST ≤18°C during the May–June period also showed significant difference among regions (one-way ANOVA: *F*_2,42_ = 27.87, *p*<0.0001), with longer periods in Catalonia (3.1±0.27) than in Sardinia (1.1±0.23) and Majorca (0.66±0.23).

## Discussion

Taken together, our results indicate that different processes acting at different spatial scales are influencing the demographic fate of the keystone herbivore *Paracentrotus lividus* in Mediterranean habitats. While regional scale factors determine larval availability and settlement patterns of the pelagic stages, once in the benthos, processes linked to local-scale habitat features become crucial in controlling the population outcome. In fact, the significance of regression analyses between life stages in rocky macroalgal and seagrass habitats diverge, indicating that habitat features play a central role in regulating the size of populations. In rocky macroalgal habitats, the major decoupling occurs between the settler and the recruit stages, forced by factors largely operating at medium and regional scales, as evidenced by the large variability associated with those scales. In the seagrass habitats most settlers failed to recruit (i.e. there was a population bottleneck), but the adult population size exceeded that of recruits, completely decoupled from the previous life stage. This increase is likely explained by a migration of adults from nearby habitats and the processes regulating such transitions are likely occurring at local scales.

Regional-scale (>100 km) factors determine the abundance of the pelagic stage of urchin life history. The abundance of larvae along the Catalan coast, while within the ranges reported in previous studies in the same region [24], [40], was about one order of magnitude higher than in Sardinia, and no larvae were found in Majorca. Several mechanisms operating at such a large spatial scale can influence the regional differences found. On the one hand, the presence of sub-basin gyres and meso-scale instability within the Balearic sub-basin [45] could prevent the dispersal of larvae via coastal and rim currents and through the mid sea (see [46] for similar effects). Moreover, the Balearic sub-basin consistently attains greater records of SST [38]–[39], which may negatively affect larval abundance, particularly because the settlement peak of *Paracentrotus lividus* larvae occurs mostly during spring when SST is ≤18°C. This window of “optimal” temperature conditions was narrower in Majorca (ca. <1 week) than in the other regions (between 1 and 3 weeks) and may constrict the period for the development of viable eggs and larvae [45]. Additionally, spring development of sea urchin larvae can also be limited by food availability when concentrations of chlorophyll *a* and particles in the plankton are low [47], such as in the extremely oligotrophic waters of both the Balearic Island sites and the Sardinian coast [48]. The large spatial scale variability found in larval stages also suggests that there is not a single larval pool shared among the three study regions. Larval pools likely remain within the same region where they have been released, probably with a low genetic connectivity among sea urchin populations across distances greater than tens of kilometers. This would be coherent with findings using genetic markers [49]–[50], suggesting that genetic flow at large spatial scales can only take place sporadically, in years of large mass spawning [51].

Abundance patterns of settlers were very similar to those observed for larvae, but with larger regional variance in rocky macroalgal vs. seagrass habitats. The number of settlers in all regions was higher on the rocks than in seagrass beds, while the magnitude of the regression coefficient between life-stages (larvae and settlers) was lower in the former. Our results suggest that larvae use active mechanisms that select rocky macroalgal beds whereas rates of benthic fixation in seagrass habitats may be determined by the trapping effect of the leaf canopy. It is known that some larvae detect chemical cues from specific substrates such as coralline algae [52]–[53], which are commonly abundant on rocky bottoms and may stimulate settlement of larvae onto these habitats. In contrast, sea urchin larvae in seagrass habitats may passively be retained, as it has been observed for sediments [54]–[55] and other larvae [56]–[57]. Hence, active selection may explain the larger abundance of settlers in the rocky macroalgal habitats and the lower relationship with larval availability, while trapping by the leaf canopy would be less efficient (lower abundance) but would result in a greater relationship between larvae and settlers' abundances.

Life-stage transitions in the benthos and the processes acting on them differ substantially between habitats. In seagrass meadows, numbers of recruits were ∼4 times lower than in the rocky macroalgal habitats, consistently with patterns from other studies [36], [58]. The decoupling between recruits and adult stages identifies an important bottleneck in seagrass habitats, whereby recruitment does not effectively contribute to the next life-stage. This pattern of reduced recruitment was consistent across all three regions, indicative of a strong relationship between the successive life-stages of larvae and recruits. Seascape-dependent mortality-recruitment relationships, like the one observed here, have been indicated as a result of selective predation by invertebrates and fish, and can scale up to influence regional traits [59]–[61]. In *P. oceanica* seagrass beds, predation pressure on small sea urchins can be very important [62], but predatory fishes are less abundant than in rocky habitats [63] and the presence of recruits seems to be mostly regulated by the availability of bare (unburied) seagrass rhizomes that may act both as a refuge from predation and as a protection against sand abrasion [36], [43]. However, the low proportion of this type of suitable substrate in most seagrass meadows [43] might explain the low recruitment success in this habitat.

In contrast, recruitment success in rocky macroalgal habitats is important, but a decoupling occurs with the previous life-stage (i.e. settlers). This decoupling is associated with an important part of medium to regional spatial scale variability that seems to shape that transition. In rocky macroalgal habitats, sea urchins, particularly small individuals, are highly susceptible to predation [62], [64]–[65] and the associated mortality is influenced by habitat complexity both directly and indirectly through the availability of refuges for urchins and their predators. In fact, the abundance of predatory fishes may vary among study sites as a result of varying distances from marine reserves [66] which could explain some variability observed at the local scale. The abundance of common predatory fishes such as *Coris julis*, *Thalassoma pavo* and *Diplodus vulgaris* [65] in Mediterranean rocky reefs is also strongly correlated with habitat complexity and heterogeneity (e.g., rugosity, number of boulders) occurring at local and sub-local spatial scales [67]. In turn, refuge provided by structural complexity, such as algal assemblages and crevices, can increase the chance of sea urchins to escape from predatory fishes at local scales [67]. Additionally, invertebrate predators such as sea stars, crustaceans, or gastropods [29], and sedentary fishes foraging on the seafloor may also induce microhabitat selection [66] that contribute to enhance variability at small spatial scales.

Among adults, a significant relationship with recruits was only observed in the rocky macroalgal habitats. In contrast, adult numbers in seagrass habitats increased substantially as compared to recruits and, on average, were similar to the adult values observed in rocky macroalgal habitats. The processes that shaped this decoupling were mostly associated with local scale (i.e. Zone) variability. We suggest migration from adjacent rocky macroalgal habitats into seagrass beds as the most reliable explanation. In fact, habitat type and landscape features have been often shown to influence the distribution of organisms both by determining the availability of shelter and by influencing faunal dispersal [25]–[26]. Habitat less suited for recruitment may receive inputs of individuals from habitats more suited for recruitment when resources (e.g. food, ground) in the latter become scarce [68]–[69]. In the case of the sea urchin *Paracentrotus lividus*, rocky substrates are recognized as a more suitable ground for sea urchin recruitment and may act as a source of migrants to adjacent seagrass beds [36], [70]–[71]. Hence, the local associated variability in seagrass habitats at this stage may be explained by the availability of functional connections across seascapes at each Site. Variations in habitat size and shape —often resulting from abiotic and/or human disturbance [72]— can affect the abundance of individuals by altering connections across habitats patches, particularly at the local and sub-local spatial scales (i.e. few meters) covered during *P. lividus* daily trips for food and refuge [73]–[74]. Once in seagrass habitats, the effect of the leaf canopy can reduce predation on young adults [62] but predation rates may be enhanced on more physically exposed transient individuals, at least until they attain certain protection in size at test diameters >4 cm [65].

To conclude, there is not a single, simple factor to explain the spatial arrangements of marine organisms, and integrative studies looking simultaneously at processes limiting and/or regulating the planktonic and benthic life-stages are needed to understand the factors shaping abundance and distribution of populations, particularly when keystone species are considered. This complexity of spatial scales and habitat-dependent processes demands a multiplicity of approaches when addressing habitat conservation actions, yet at present such actions are largely focused on management of predation processes and fish numbers. Multiple factors (e.g., eutrophication and temperature) can influence larval abundance and distribution and, subsequently, settlement success. In rocky macroalgal habitats, predation is still the most likely mechanism controlling adult populations, and as such, management of fish communities constitutes a valid approach. In seagrass meadows, however, other processes such as human disturbance, heterogeneity in habitat structure and landscape connections may be the main mechanisms influencing the abundance and spatial patterns of sea urchins by disrupting the mobility patterns of organisms at various life stages. Management needs to broaden its view beyond predation and include the landscape perspective and water-quality aspects that, in combination with predation, regulate populations of this keystone herbivore.
