# Correction: Habitat and Scale Shape the Demographic Fate of the Keystone Sea Urchin *Paracentrotus lividus* in Mediterranean Macrophyte Communities

**DOI:** 10.1371/annotation/7e978cb1-df3b-42f1-94d9-fcc424cf7167

**Published:** 2012-07-09

**Authors:** Patricia Prado, Fiona Tomas, Stefania Pinna, Simone Farina, Guillem Roca, Giulia Ceccherelli, Javier Romero, Teresa Alcoverro

The following information was missing from the Funding section: JR was funded by the project CTM2010-22273-c02-01.

